# An EGFR/HER2-Bispecific and Enediyne-Energized Fusion Protein Shows High Efficacy against Esophageal Cancer

**DOI:** 10.1371/journal.pone.0092986

**Published:** 2014-03-24

**Authors:** Xiao-Fang Guo, Xiao-Fei Zhu, Wan-Cai Yang, Sheng-Hua Zhang, Yong-Su Zhen

**Affiliations:** 1 Department of Microbiology, Xinxiang Medical University, Xinxiang, China; 2 School of Laboratory Medicine, Xinxiang Medical University, Xinxiang, China; 3 Department of Pathology, Xinxiang Medical University, Xinxiang, China; 4 Department of Pathology, University of Illinois at Chicago, Chicago, United States of America; 5 Institute of Medicinal Biotechnology, Chinese Academy of Medical Sciences and Perking Union Medical College, Beijing, China; University of Central Florida, United States of America

## Abstract

Esophageal cancer is one of the most common cancers, and the 5-year survival rate is less than 10% due to lack of effective therapeutic agents. This study was to evaluate antitumor activity of Ec-LDP-Hr-AE, a recently developed bispecific enediyne-energized fusion protein targeting both epidermal growth factor receptor (EGFR) and epidermal growth factor receptor 2 (HER2), on esophageal cancer. The fusion protein Ec-LDP-Hr-AE consists of two oligopeptide ligands and an enediyne antibiotic lidamycin (LDM) for receptor binding and cell killing, respectively. The current study demonstrated that Ec-LDP-Hr had high affinity to bind to esophageal squamous cell carcinoma (ESCC) cells, and enediyne-energized fusion protein Ec-LDP-Hr-AE showed potent cytotoxicity to ESCC cells with differential expression of EGFR and HER2. Ec-LDP-Hr-AE could cause significant G2-M arrest in EC9706 and KYSE150 cells, and it also induced apoptosis in ESCC cells in a dosage-dependent manner. Western blot assays showed that Ec-LDP-Hr-AE promoted caspase-3 and caspase-7 activities as well as PARP cleavage. Moreover, Ec-LDP-Hr-AE inhibited cell proliferation via decreasing phosphorylation of EGFR and HER2, and further exerted inhibition of the activation of their downstream signaling molecules. *In vivo*, at a tolerated dose, Ec-LDP-Hr-AE inhibited tumor growth by 88% when it was administered to nude mice bearing human ESCC cell KYSE150 xenografts. These results indicated that Ec-LDP-Hr-AE exhibited potent anti-caner efficacy on ESCC, suggesting it could be a promising candidate for targeted therapy of esophageal cancer.

## Introduction

Esophageal cancer is one of the most common cancers, as well as the sixth most common cause of cancer-related deaths in the world. The northern regions in Henan province of China have the highest incidence of esophageal cancer, particularly the esophageal squamous cell carcinoma (ESCC) [1], [2]. Though many treatment options are now available, including surgery, combined modality strategies such as pre- or post-operation chemotherapy with or without radiation, and definitive chemoradiation, the prognosis of esophageal cancer is poor with a 5-year survival rate less than 10% [3]–[5]. Due to the disadvantages of chemotherapy agents, such as severe toxic side-effects, the high incidence of drug-resistance, and little effects on survival, there is an urgent need for the development of novel effective therapeutic agents, targeting specific molecular abnormalities in esophageal carcinomas, especially those in the human epidermal growth factor receptor (HER) family [6].

The HER family of tyrosine kinase receptors contains four members: HER1/EGFR, HER2/neu, HER3 and HER4. Ligand binding to the receptors results in receptor dimerization, then initiates a series of intracellular events that eventually promote cell growth, proliferation, differentiation and migration [7]. Overexpression of epidermal growth factor receptor (EGFR) and human epidermal growth factor receptor 2 (HER2) has been observed in many human cancers, such as lung, head and neck, breast, ovary, and they have been shown to play important roles in both formation and progression of many commonly occurring cancers [8]. In esophageal cancer, EGFR overexpression occurs in 30%–90% [9], [10], and HER2 overexpression ranges from 19%–43% [11]. Furthermore, overexpression of both EGFR and HER2 was observed in 18%–25% patients with esophageal cancer [12], [13]. Therefore, an agent targeting both EGFR and HER2 would exhibit more effective therapeutic effects on esophageal cancer patients.

Ec-LDP-Hr-AE, a bispecific fusion protein consisting of two oligopeptides (Ec, 22 amino acids of EGF and Hr, VH CDR3 region of anti-HER2 C6.5 antibody) specific for EGFR and HER2, and an enediyne antibiotic lidamycin (LDM), was constructed and reported in our previous study [14]. It showed potent cytotoxicity to a variety of carcinoma cells *in vitro*, and was highly effective in inhibiting the growth of human ovarian caner SK-OV-3 xenografts *in vivo*. However, the antitumor efficacy of Ec-LDP-Hr-AE on esophageal cancer is not well studied. In this study, we not only measured the binding affinity of Ec-LDP-Hr fusion protein to esophageal caner cells, but also evaluated the potency of energized fusion proteins *in vitro* and *in vivo*. The effects of Ec-LDP-Hr-AE on cell cycle distribution and apoptosis were also assayed. To elucidate the mechanisms involved in the cytotoxicity and apoptosis-induction of Ec-LDP-Hr-AE, the expression of apoptosis related molecules and key molecules in the HER signaling pathways were analyzed as well.

## Materials and Methods

### Ethics statement

Female BALB/c nude mice (6–8 weeks) used in the experiments were purchased from the Institute of Laboratory Animal Sciences, Chinese Academy of Medical Sciences, and hosted under specific pathogen-free conditions. The experimental protocol was approved by the Animal Experiments Ethics Committee of Institute of Medicinal Biotechnology, Chinese Academy of Medical Sciences.

### Cell lines and culture

Human esophageal carcinoma cell lines EC1, ECa109, EC9706 and KYSE150 and mouse fibroblast cell line NIH 3T3 were obtained from Cell Center of Peking Union Medical College, China, and cultured under a humidified atmosphere of 5% CO_2_ at 37°C in RPMI 1640 supplemented with 10% fetal bovine serum (FBS, Gibco, Carlsbad, CA, USA), 2 mmol/L glutamine, 100 U/ml penicillin and 100 μg/ml streptomycin.

### Reagents and antibodies

(3-(4, 5-dimethyl-thiazol-2-yl)-2, 5-dipheny-ltetrazolium bromide (MTT), fluorescein isothiocyanate (FITC) and isoprophyl-β-D-thiogalactopranoside (IPTG) were purchased from Sigma Aldrench Chemical Inc. (St. Louis, MO, USA). All the antibodies including phosphorylated-HER2, -EGFR, -AKT, -extracellular regulated protein kinase (ERK), -p38 mitogen-activated protein kinase (MAPK), -c-Jun N-terminal kinase (JNK) monoclonal antibodies and anti-EGFR, -HER2, -AKT, -ERK, -p38MAPK, -JNK, -caspase 3, -caspase 7, -cleaved PARP antibodies were obtained from Cell Signaling Technology (Danvers, MA, USA). Anti-β-actin antibodies were purchased from Santa Cruz Biotechnology (Santa Cruz, CA, USA). Horseradish peroxidase (HRP)-conjugated goat anti-rabbit/mouse antibodies were also purchased from by Cell Signaling Technology.

### Preparation of energized fusion proteins

The preparation of bispecific fusion protein Ec-LDP-Hr-AE and its corresponding monospecific fusion proteins Ec-LDP-AE and LDP-Hr-AE were described in our previous study [14]. Briefly, DNA fragments coding for Ec-LDP-Hr, Ec-LDP and LDP-Hr were obtained by PCR and DNA cloning techniques, and then they were inserted into pET30a vector to generate the expression plasmids pET-*Ec-LDP-Hr*, pET-*Ec-LDP* and pET-*LDP-Hr*. These plasmids were then transformed into *Escherichia coli BL21*(*DE3*), and the fusion proteins were expressed by addition of IPTG. The fusion proteins were extracted from the periplasmic space of *E.coli* by osmotic shock method (pET system manual, 9^th^ edition, Novagen) and purified by affinity chromatography (HisTrap HP column, GE Healthcare). The energized fusion proteins Ec-LDP-Hr-AE, Ec-LDP-AE and LDP-Hr-AE were constructed by integrating the active enediyne chromophore (AE) of lidamycin into the Ec-LDP-Hr, Ec-LDP and LDP-Hr proteins, respectively.

### Binding affinity assay

A flow-cytometry-based immunofluorescence assay was used to measure the binding affinity of fusion protein Ec-LDP-Hr to esophageal cancer cells [15]. The Ec-LDP-Hr protein was FITC labeled for 16 h in a carbonate buffer solution (100 mmol/L NaHCO_3_, 10 mmol/L Na_2_CO_3_, pH 9.0) at 4°C. Labeled protein was separated from unbound FITC by using Sephadex G-25 column (GE Healthcare). Then the FITC-labeled Ec-LDP-Hr protein was incubated with 10^6^ EC9706 cells, KYSE150 cells or NIH 3T3 cells in a 100 μl volume of buffer (PBS+2%FBS) for 2 h at room temperature. Following three washes with 500 μl of buffer, cells were analyzed with flow cytometer (BD Company). The data were analyzed with Prism 5 software (GraphPad Software).

### MTT assay

Cells were detached by trypsinization and plated in 96-well flat-bottomed plates, cultured for 24 h before exposure to various concentrations of LDM, Ec-LDP-Hr-AE, Ec-LDP-AE or LDP-Hr-AE for 48 h. MTT solution (5 mg/ml, 20 μl) was added to each well and incubated for another 4 h at 37°C. The supernatant was removed and 150 μl DMSO was added to each well. The absorbance at 570 nm was measured using a Multiskan Spectrum instrument (Thermo Labsystems, Rochford, IL, USA). Absorbance values were expressed as a percentage of that for untreated cells, and the concentrations of tested agents resulting in 50% growth inhibition (IC_50_) were calculated.

### Cell cycle distribution analysis

The effects of bispecific fusion protein Ec-LDP-Hr-AE on cell cycle were evaluated using propidium iodide (PI) staining. After treatment with 0.1 nmol/L, 0.5 nmol/L and 1 nmol/L Ec-LDP-Hr-AE for 48 h, EC9706 and KYSE150 cells were digested by trypsin-EDTA and washed with PBS. The cells were then resuspended in 500 μl PBS with 50 μg/ml PI and 100 μg/ml RNase A. After incubation at 37°C for 30 min, cells were analyzed for fluorescence with a flow cytometer (BD Company).

### Cell apoptosis assay

The effects of bispecific fusion protein Ec-LDP-Hr-AE on inducing apoptosis in ESCC cells were measured by using Hoechst staining and Annexin V-FITC/PI staining. For Hoechst staining, KYSE150 cells were grown on coverslides and incubated for 24 h, then 0.1 nmol/L, 0.5 nmol/L and 1 nmol/L Ec-LDP-Hr-AE were added and incubated for another 48 h. Cells in coverslides were fixed with methanol, washed with PBS, and stained by 1 mg/ml Hoechst 33342 for 15 min. The images were observed under a fluorescence microscope (Nikon TE 2000 u). For Annexin V-FITC/PI staining, the apoptotic cells were measured by an Annexin V-FITC/PI staining kit (Biosea technology). After 0.1 nmol/L, 0.5 nmol/L and 1 nmol/L of Ec-LDP-Hr-AE treatment for 48 h, cells were harvested, washed twice with PBS, and centrifuged at 1,000 rpm for 5 min. The cell pellets were resuspended in 500 μl binding buffer containing of 10 μl Annexin V-FITC and 5 μl PI, incubated at room temperature for 15 min, and then analyzed for fluorescence with a flow cytometer (BD Company).

### Western blot analysis

Cells were lysed for 30 min in radioimmunoprecipitation assay (RIPA) buffer contained several protease inhibitors (e.g. 1 μg/ml aprotinin, 10 μg/ml leupeptin, 1 mmol/L phenylmethylsulfonyl fluoride, 2 mmol/L NaVO_4_, and 50 mmol/L NaF). Protein extracted from cells was quantitated using bicinchoninic acid kit (Pierce Biochemicals), and 30 μg of each total protein were applied on 10% SDS-PAGE and then electroblotted onto polyvinylidene difluoride membranes (Millipore). The membranes were incubated with 1% BSA for 2 h at room temperature before incubation overnight at 4°C with primary antibodies (diluted 1∶1000 with TBST buffer, Cell Signaling Technology). Then the membranes were incubated with secondary HRP-conjugated antibodies (1∶5000 dilution; Cell Signaling Technology) for 1 h after washing three times with TBST buffer. The specific bands were visualized with the Immobilon Western Chemiluminescent HRP Substrate kit (Millipore) and captured by ChemiImager 5500 imaging system (Alpha Innotech Corp.).

### *In vivo* efficacy assay

*In vivo* efficacy of energized fusion proteins was evaluated in a KYSE150 xenografts nude mouse model. 1×10^7^ KYSE150 cells suspended in 200 μl PBS were inoculated s.c. in right armpit of nude mice. After 3 weeks, the tumors were taken out from nude mice and dissected aseptically in the sterile saline. The pieces of tumor tissue (2 mm^3^ in size) were then transplanted into the right armpit of nude mice by a trocar, and the wound was sealed by collodion. Tumor-bearing mice were randomly divided into 3 groups (n = 7) when the tumor size was over 100 mm^3^ (about 10 days later). Lidamycin (0.05 mg/kg) and bispecific fusion protein Ec-LDP-Hr-AE (0.3 mg/kg) were injected i.v. in the tail vein and given in a 200 μl volume of PBS at day 11 and day 21, respectively. Control group of mice received 200 μl PBS treatment at the same time as fusion proteins. Tumor size was measured every 3 day and tumor volume was determined by length × width^2^/2. The inhibition rates were calculated by 1 − tumor volume (treated)/tumor volume (control) × 100%.

### Statistical analysis

Results of quantitative data in this study were presented as the mean ± SD. Differences between groups were statistically analyzed using unpaired two-tailed t-test, and P-values <0.05 were considered statistically significant.

## Results

### Preparation of energized fusion proteins

According to our previous approach, the genes coding for fusion proteins Ec-LDP-Hr, Ec-LDP, and LDP-Hr were constructed and the encoded proteins were expressed in the periplasmic space of *E.coli*. Fusion proteins were purified by Ni^2+^ affinity chromatography and the purity of fusion proteins was all over 90% as determined by SDS-PAGE and high-performance-liquid-chromatography (HPLC). The active enediyne chromophore (AE) of LDM and three fusion proteins were reconstituted *in vitro* to generate the energized fusion proteins Ec-LDP-Hr-AE, Ec-LDP-AE and LDP-Hr-AE. The results from reverse-phase HPLC showed that three energized fusion proteins were successfully assembled.

### Binding affinity of Ec-LDP-Hr protein to esophageal cancer cells

**Figure 1:**
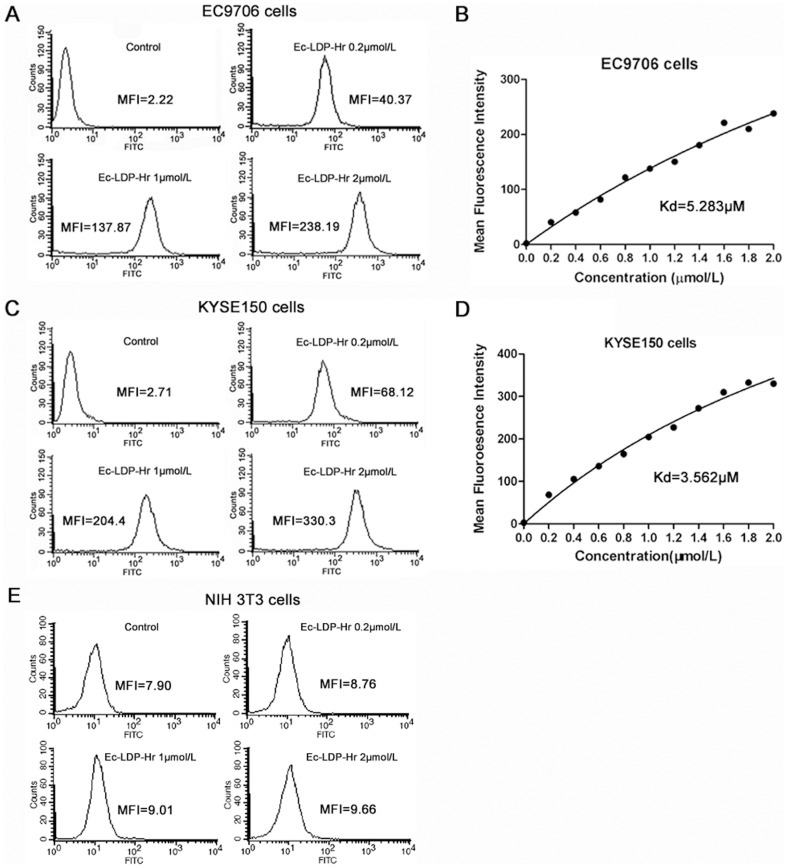
Binding affinity of Ec-LDP-Hr fusion protein to esophageal cancer cells. EC9706 cells (A), KYSE150 cells (C) or NIH 3T3 cells (E) were incubated with FITC-labeled Ec-LDP-Hr protein at indicated concentrations, and the mean fluorescence intensities (MFIs) were analyzed by flow cytometer. Increased concentrations of FITC-labeled Ec-LDP-Hr proteins were incubated with EC9706 cells (B) or KYSE150 cells (D). Following FACS, the MFIs were plotted versus protein concentrations.

EC9706 cells, KYSE150 cells and NIH 3T3 cells were incubated with various concentrations of FITC-labeled Ec-LDP-Hr, and the fluorescence intensities were measured by flow cytometer. The mean fluorescence intensities (MFIs) of EC9706 cells and KYSE150 cells both significantly increased (P<0.05), which indicated Ec-LDP-Hr had strong binding activity to the esophageal cancer cells (Fig 1A, 1C). However, the MFIs of NIH 3T3 cells did not show significant increase, which meant Ec-LDP-Hr was unable to bind to EGFR and HER2 negative NIH 3T3 cells (Fig 1E). According to the MFIs and the Ec-LDP-Hr concentrations, the binding affinity (K_d_) values were calculated with Prism 5 software. The K_d_ values for Ec-LDP-Hr bound to EC9706 and KYSE150 cells were 5.283 μmol/L and 3.562 μmol/L, respectively (Fig. 1B, 1D).

### Cytotoxicity of energized fusion proteins *in vitro*

**Figure 2:**
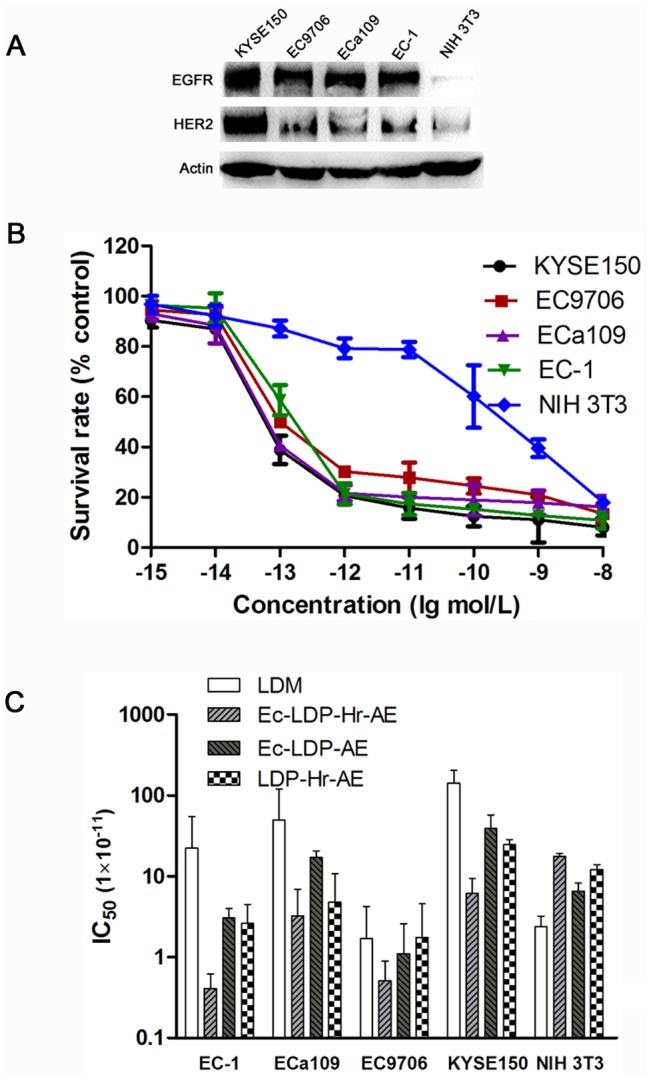
Cytotoxicity of Ec-LDP-Hr-AE on esophageal cancer cells. (A) Expression of EGFR and HER2 on different esophageal cancer cells and NIH 3T3 cells analyzed by Western blot. (B) The cell killing effects of Ec-LDP-Hr-AE on esophageal cancer cell lines KYSE150, EC9706, ECa109 and EC-1and mouse fibroblast cell line NIH 3T3 were tested by MTT assays. Cells were exposed to various concentrations of Ec-LDP-Hr-AE for 48 h and the results were obtained from three independent experiments. (C) The IC_50_ values of lidamycin (LDM), Ec-LDP-Hr-AE, Ec-LDP-AE, and LDP-Hr-AE against 4 kinds of esophageal cancer cells and NIH 3T3 cells were measured by MTT assay. Columns, mean of triplicate experiments, bars, SD.

The cytotoxicity of bispecific energized fusion protein Ec-LDP-Hr-AE was carried out on four human esophageal squamous cell carcinoma (ESCC) cell lines expressing different levels of EGFR and HER2 and the EGFR/HER2 negative NIH 3T3 cells by using MTT assays (Fig. 2A). LDM and monospecific energized fusion proteins Ec-LDP-AE and LDP-Hr-AE were also tested for comparison. As shown in Fig. 2B, the bispecific energized fusion protein Ec-LDP-Hr-AE killed both ESCC cells and NIH 3T3 cells with very high potency. The IC_50_ values of Ec-LDP-Hr-AE for 4 ESCC cells were below the 10^−10^ mol/L level (Fig. 2C). However, the bispecific Ec-LDP-Hr-AE protein was not always more potent than the monospecific counterparts and LDM. Results from statistical analysis revealed that the differences in IC_50_ values of LDM, Ec-LDP-Hr-AE, Ec-LDP-AE and LDP-Hr-AE for EC-1, ECa109 and EC9706 cells were not statistically significant. But the differences in IC_50_ values for KYSE150 cells were significant (P<0.05). Furthermore, IC_50_ value of Ec-LDP-Hr-AE for NIH 3T3 cells did not show significant differences when compared with the four ESCC cells.

### Effects of bispecific fusion protein Ec-LDP-Hr-AE on cell cycle distribution

**Figure 3:**
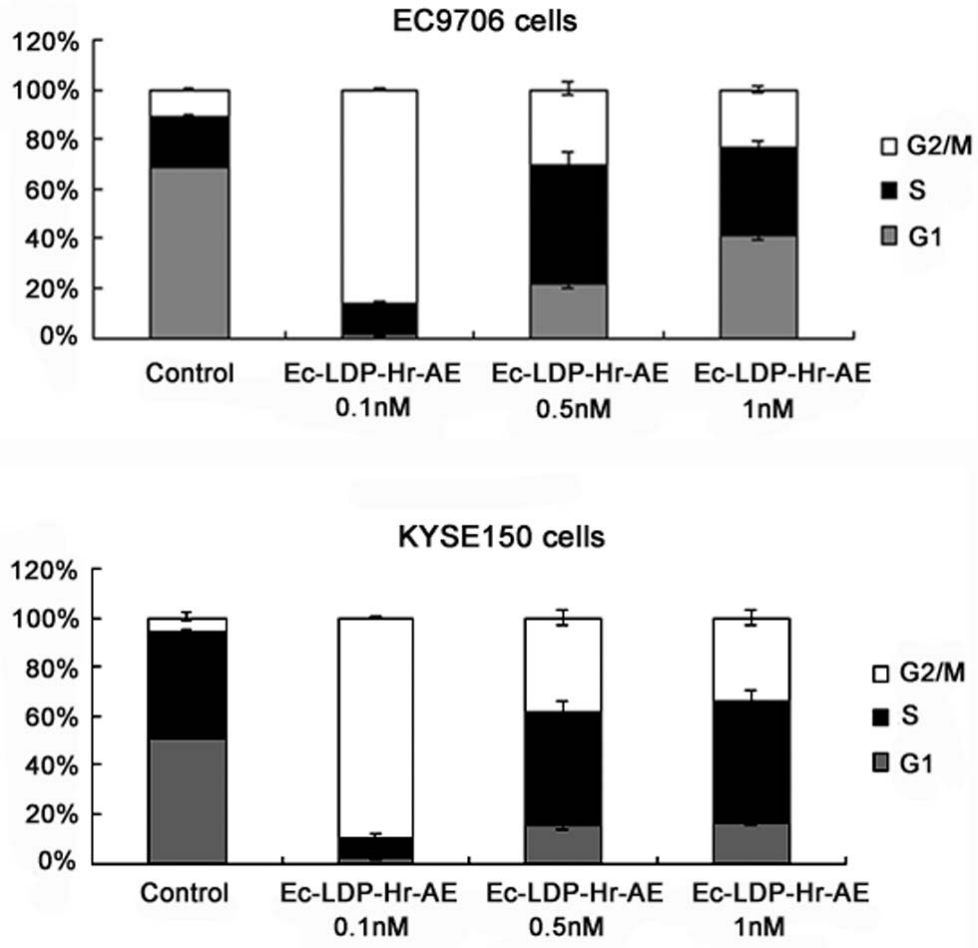
Cell cycle distribution of esophageal cancer cells after treatment with Ec-LDP-Hr-AE. EC9706 cells or KYSE150 cells were exposed to Ec-LDP-Hr-AE for 48 h at the indicated concentrations and cell cycle distribution was determined by flow cytometry after PI staining. Columns, mean of triplicate experiments, bars, SD.

EC9706 and KYSE150 cells were exposed to 0.1, 0.5 and 1 nmol/L of Ec-LDP-Hr-AE for 48 h, and the cell cycle distribution was evaluated by PI staining and flow cytometry analysis. Control cells (EC9706 and KYSE150) distributed in G2-M phase were 10.51%±0.98% and 6.26%±1.96% respectively, whereas cells treated with 0.1 nmol/L of Ec-LDP-Hr-AE distributed in G2-M phase were 86.00%±0.98% and 89.36%±0.71% respectively. These data indicated that a significant G2-M arrest was caused by Ec-LDP-Hr-AE treatment (Fig. 3). Although there was a great shift (about 12.84%∼31.53% increases) into G2-M phase after exposure to 0.5 nmol/L and 1 nmol/L of Ec-LDP-Hr-AE, the cells distributed in G2-M phase were less than that of treated with 0.1 nmol/L of Ec-LDP-Hr-AE, which indicated the G2-M phase cells reached the peak with 0.1 nmol/L of Ec-LDP-Hr-AE treatment (Fig. 3).

### Effects of bispecific fusion protein Ec-LDP-Hr-AE on apoptosis

**Figure 4:**
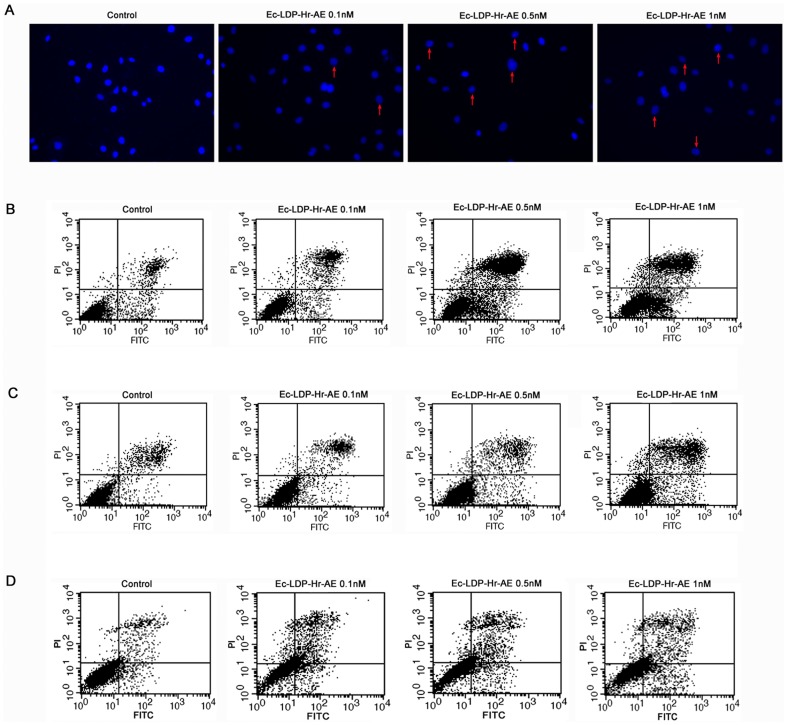
The effects of apoptosis-induction by Ec-LDP-Hr-AE treatment. (A) KYSE150 cells were treated by Ec-LDP-Hr-AE at indicated concentrations for 48 h, and then stained by Hoechst 33342. The images were observed under a fluorescence microscope at ×200. EC9706 cells (B), KYSE150 cells (C) or NIH 3T3 cells (D) were exposed to the indicated concentrations of Ec-LDP-Hr-AE for 48 h. Cells were harvested and stained with a combination of FITC-Annexin V and PI. The lower left quadrants (FITC^−^/PI^−^) indicated the viable cells, and the lower right quadrants (FITC^+^/PI^−^) indicated the early apoptotic cells. The upper right quadrants (FITC^+^/PI^+^) indicated the late apoptotic cells, and the upper left quadrants (FITC^−^/PI^+^) indicated the dead cells.

The results from Hoechst 33342 staining and Annexin V-FITC/PI staining assays revealed that apoptotic cells (EC9706 and KYSE150) increased markedly in a dosage-dependent manner after treatment with Ec-LDP-Hr-AE. The Hoechst 33342 staining was used to detect the changes in nuclear morphology. The nuclei of untreated cells were normal in appearance and exhibited diffused staining of the chromatin. After exposure to different concentrations of Ec-LDP-Hr-AE for 48 h, KYSE150 cells presented typical morphological changes of apoptosis such as chromatin condensation or a shrunken nucleus (Fig. 4A). As shown in Fig. 4B, the ratios of apoptotic EC9706 cells after treatment with 0.1, 0.5 and 1 nmol/L of Ec-LDP-Hr-AE were 15.06%±0.29%, 38.10%±0.64% and 50.00%±0.39% respectively, which showed significant increases compared with control cells (P<0.01). The apoptotic cells also increased a lot for the KYSE150 cells after exposure to 0.1, 0.5 and 1 nmol/L of Ec-LDP-Hr-AE, with the apoptosis ratios of 16.29%±0.35%, 21.54%±0.51% and 32.99%±0.38%, respectively (P<0.01 versus control, Fig. 4C). In addition, Ec-LDP-Hr-AE induced apoptosis in EGFR/HER2 negative NIH 3T3 cells, and the ratios of apoptotic cells after treatment with 0.1, 0.5 and 1 nmol/L of Ec-LDP-Hr-AE were 16.47%±0.44%, 15.28%±0.45% and 19.90%±0.12%, respectively. The apoptotic NIH 3T3 cells were significantly less than that of EC9706 cells and KYSE150 cells at the exposure dosages of 0.5 nmol/L and 1 nmol/L of Ec-LDP-Hr-AE (P<0.05, Fig. 4D).

### Effects of bispecific fusion protein Ec-LDP-Hr-AE on EGFR/HER2 signaling pathways activation

**Figure 5:**
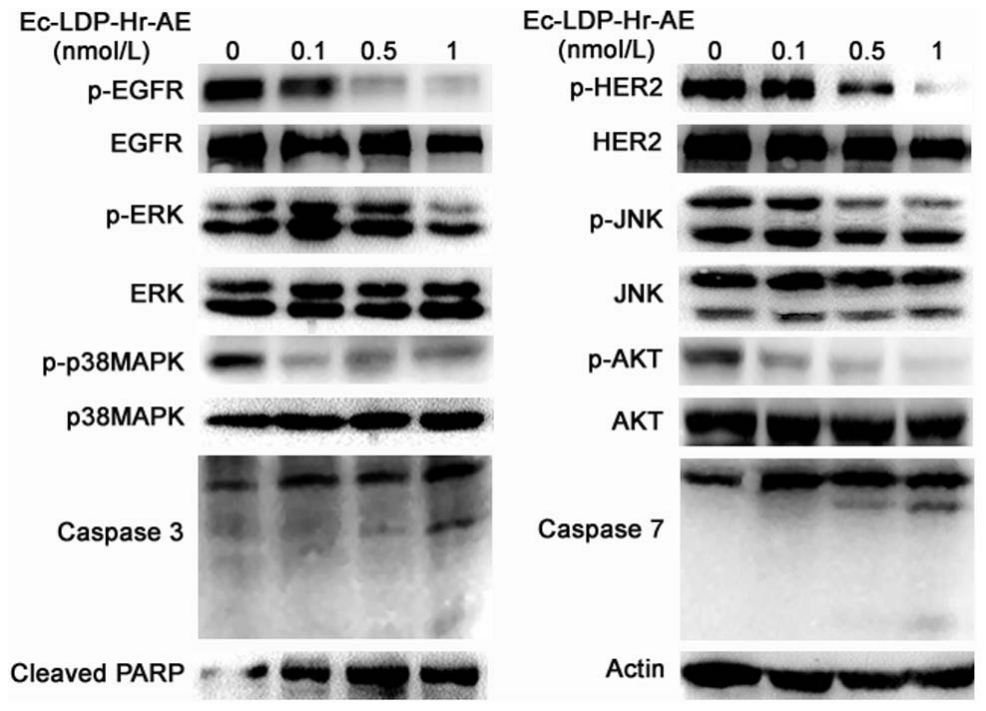
Effects of Ec-LDP-Hr-AE on the EGFR/HER2 signaling. The expression of apoptosis related molecules (e.g. Caspase 3, Caspase 7 and PARP) and key molecules in the EGFR/HER2 signaling pathways (e.g. phosphorylated-EGFR, -HER2, -ERK, -p38MAPK, -JNK, -AKT) were determined by Western blot analysis. β-actin was served as a loading control.

To characterize the molecular mechanisms involved in the cytotoxicity and apoptosis-induction of Ec-LDP-Hr-AE, we examined its effects on the expression of apoptosis related molecules and several key molecules in the EGFR/HER2 signaling pathways. As shown in Fig. 5, Ec-LDP-Hr-AE promoted caspase-3 and caspase-7 activities as well as PARP cleavage, indicating that Ec-LDP-Hr-AE-induced apoptosis may be associated with mitochondrial pathways. Moreover, treatment with Ec-LDP-Hr-AE leaded to significant decreased phosphorylation of EGFR and HER2, whereas the expression of inactivated EGFR and HER2 were not changed. The phosphorylation of downstream signaling molecules of EGFR/HER2 pathway, such as AKT, ERK, p38MAPK and JNK was further inhibited by the treatment of Ec-LDP-Hr-AE (Fig. 5). The densitometry data demonstrated that the phosphorylated HER2 and p38MAPK decreased significantly with all indicated concentrations of Ec-LDP-Hr-AE treatment (data not shown, P<0.05). Phosphorylated EGFR, AKT and JNK decreased significantly with 0.5 nmol/L and 1 nmol/L of Ec-LDP-Hr-AE treatment (P<0.05) and phosphorylated ERK decreased significantly with 1 nmol/L of Ec-LDP-Hr-AE treatment (data not shown, P<0.05). The levels of total AKT, ERK, p38MAPK and JNK were not affected by the treatment of Ec-LDP-Hr-AE, and the densitometry data supported this conclusion (data not shown).

### *In vivo* efficacy of energized fusion proteins

**Figure 6:**
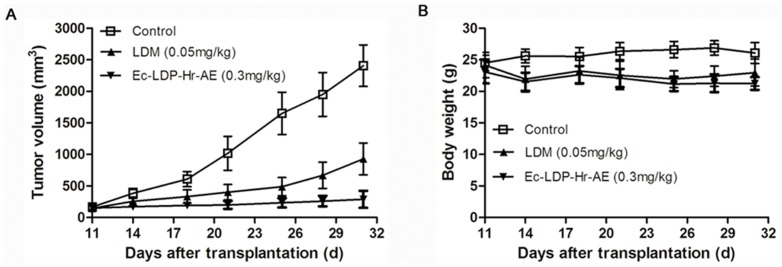
*In vivo* inhibitory effects of lidamycin (LDM) and Ec-LDP-Hr-AE. Nude mice (n = 7) bearing human esophageal carcinoma KYSE150 xenografts were treated with LDM or Ec-LDP-Hr-AE on day 11 and day 21 after tumor inoculation by tail vein injection. The mean tumor volumes (A) and the mean body weights of mice (B) in each group are shown. Ec-LDP-Hr-AE at the dose of 0.3 mg/kg inhibited the growth of KYSE150 xenografts by 88%, which showed statistically significant differences compared with LDM treated group at the maximum tolerated dose (0.05 mg/kg) (P<0.05).

*In vivo* antitumor effects of energized fusion proteins were evaluated in a KYSE150 xenografts nude mouse model. As shown in Figure 6A, LDM suppressed the growth of KYSE150 xenografts by 61.4% at the maximum tolerated dose (0.05 mg/kg), and the bispecific fusion protein Ec-LDP-Hr-AE at the dose of 0.3 mg/kg inhibited the growth of KYSE150 xenografts by 88%, which showed statistically significant differences (P<0.05) compared with LDM-treated group at 0.05 mg/kg. No animal deaths were found in the treated groups, and the curves of body weight indicated that the animals tolerated well to the administered dose of Ec-LDP-Hr-AE (Fig. 6B).

## Discussion

Surgery, radiotherapy and chemotherapy are still the mainstay of treatment for esophageal cancer, but recently, a number of targeted therapies are being studied with the goal of improving response rate and survival in patients with esophageal cancer [6], [16], [17]. As is known, overexpression of EGFR and HER2 has been observed in over 30% of esophageal carcinomas, and their overexpression is correlated with reduced survival, increased risk of recurrence, distant metastasis, and resistance to radiotherapy [18]. Therefore, the effects of some monospecific antibodies and tyrosine kinase inhibitors that block EGFR or HER2 function, such as cetuximab, trastuzumab, gefitinib, and erlotinib on esophageal carcinoma have been examined, but the efficacy is limited so far [19]–[22]. Different from monoclonal antibodies and tyrosine kinase inhibitors, immunotoxins are considered to be highly effective on cancer therapy with the advantage of the specificity of antibodies or ligands and the cytotoxicity of toxins [23]. However, clinical trial data on the immunotoxins have shown inconsistent results. Several immunotoxins were very effective against hematologic malignancies. For example, in the phase III trial in patients with cutaneous T-cell lymphoma (CTCL), 30% of the 71 patients treated with denileukin diftitox (DAB_389_IL2, Ontak) had an objective response, including 10% complete remissions. And in a phase I trial in 31 patients with hairy cell leukemia, BL22 induced 19 complete responses (61%) and 5 partial responses (19%) [24], [25]. But for solid tumors, immunotoxins showed limited antitumor efficacy. The reasons for unsuccessful treatment of solid tumors included poor penetration into tumors and the severe immune responses [26]–[28]. Vallera and co-workers have developed novel bispecific molecules by fusing two distinct targeting ligands to a single toxin with the aim to improve specificity, and the results demonstrated that bispecific molecules showed either enhanced antitumor activity or broader spectrum of reactivity than the monospecific molecules [15], [29], [30]–[34]. The fusion protein Ec-LDP-Hr-AE we constructed previously is a bispecific molecule that targeting both EGFR and HER2. Ec-LDP-Hr-AE employs two oligopeptides for receptor binding and subsequent intracellular delivery of an enediyne antibiotic lidamycin for cell killing. In this study, the antitumor activity of Ec-LDP-Hr-AE on esophageal cancer was investigated, because co-overexpresssion of EGFR and HER2 was observed in a majority of esophageal squamous carcinomas.

Binding with corresponding receptors is the prerequisite for Ec-LDP-Hr-AE to exert its tumor cell-selective cytotoxic effects. Therefore, the binding capacity of Ec-LDP-Hr protein to ESCC cells was evaluated by using a flow-cytometry-based immunofluorescence assay. The results showed that Ec-LDP-Hr protein was able to bind to ESCC cells with high affinity. However, Ec-LDP-Hr failed to show binding activity to EGFR and HER2 negative NIH 3T3 cells. In cell viability assay, the bispecific and enediyne-energized fusion protein Ec-LDP-Hr-AE showed extremely potent killing effects on esophageal cancer cells with IC_50_ values at very low level (<10^−10^ mol/L). For the KYSE150 cells, which express both EGFR and HER2, Ec-LDP-Hr-AE was the most cytotoxic to esophageal cancer cells when comparing with LDM and two monospecific fusion proteins (Ec-LDP-AE and LDP-Hr-AE). Enhanced activity of bispecific fusion protein may be explained by that Ec-LDP-Hr-AE bound to both EGFR and HER2, and made it less likely to dissociate from the cell surface, thereby increasing the chances for internalization of its cytotoxic moiety and exerting cell killing effects. However, the bispecific Ec-LDP-Hr-AE protein was not always more potent than the monospecific counterparts and LDM as the results of statistical analysis revealed that the differences in IC_50_ values of LDM, Ec-LDP-Hr-AE, Ec-LDP-AE and LDP-Hr-AE for EC-1, ECa109 and EC9706 cells were not statistically significant. Furthermore, we found that the cytotoxicity of energized fusion proteins to ESCC cells was not correlated well with the EGFR and HER2 expression levels. For example, the IC_50_ value of Ec-LDP-Hr-AE for EC9706 cells with low HER2 expression was 5.12×10^−12^ mol/L, whereas IC_50_ value for KYSE150 cells with high EGFR and HER2 level was 6.10×10^−11^ mol/L. The similar results were also observed between the potency of monospecific fusion proteins and the EGFR/HER2 levels. This phenomenon has been observed in other studies about targeted drugs, such as lapatinib [35], [36]. As a result, further studies focusing on revealing the molecular mechanisms of sensitivity to Ec-LDP-Hr-AE are clearly needed, and this may provide useful data for selecting patients who will benefit from EGFR/HER2-bispecific agents.

To elucidate the mechanisms of bispecific Ec-LDP-Hr-AE exhibited cytotoxicity on esophageal cancer cells, PI staining, Hoechst staining, and Annexin V-FITC/PI staining studies were performed to examine cell cycle arrest and apoptosis. Results from cell cycle analysis demonstrated that Ec-LDP-Hr-AE caused significant G2-M arrest, in which the G2-M phase cells reached the peak with 0.1 nmol/L of Ec-LDP-Hr-AE treatment. This result was potentially due to the apoptotic and dead cells increased after treatment with 0.5 nmol/L and 1 nmol/L Ec-LDP-Hr-AE. Therefore, the G2-M arrest was less significant than that of 0.1 nmol/L Ec-LDP-Hr-AE treatment. Ec-LDP-Hr-AE also induced apoptosis in EC9706 and KYSE150 cells in a dosage-dependent manner, and the apoptosis induced by Ec-LDP-Hr-AE may be associated with mitochondrial pathways, because caspase-3 and caspase-7 activities as well as PARP cleavage increased significantly as shown by the Western blot analysis. Although Ec-LDP-Hr-AE induced apoptosis in the NIH 3T3 cells, the ratios of apoptotic cells were significantly less than those in KYSE150 cells or EC9706 cells after exposure to 0.5 nmol/L and 1 nmol/L of Ec-LDP-Hr-AE. These findings suggested that Ec-LDP-Hr-AE employs two oligopeptides for receptor binding and subsequent intracellular delivery of the active enediyne chromophore (AE) of lidamycin for cell killing via the way of receptor-mediated endocytosis. Based on the data of binding studies, MTT assays, and apoptosis studies, we found that receptor-mediated endocytosis might be the main way of cytotoxic AE molecules into the cells, but it was not the only way. Other non-specific mechanisms of AE molecules entry to the cells may exist. Ec-LDP-Hr-AE inhibited esophageal cancer cell proliferation by regulating the activities of EGFR and HER2 signaling pathways. It decreased the phosphorylation of EGFR and HER2, and further exerted inhibition of the activation of their downstream signaling molecules, such as AKT, ERK, p38MAPK and JNK. *In vivo*, Ec-LDP-Hr-AE significantly suppressed the growth of human esophageal cancer KYSE150 xenografts at the dose of 0.3 mg/kg with an inhibition rate of 88%. It showed statistically significant differences (P<0.05) when compared to LDM-treated group at 0.05 mg/kg tolerated dose (inhibition rate, 61.4%). Furthermore, seven mice in 0.3 mg/kg Ec-LDP-Hr-AE treatment group were all alive at the end of the experiment, and the weight loss of mice in this group did not exceed 10% of the pretreatment weight, which meant the dosage of 0.3 mg/kg of Ec-LDP-Hr-AE was well tolerated. This dosage was six times than that of LDM. These data suggested that comparing with “naked” LDM, the bispecific fusion protein showed elevated antitumor efficacy and decreased toxicity. Since the two oligopeptides specific for EGFR and HER2 were involved in the fusion protein Ec-LDP-Hr-AE, it was likely bound to tumor cells overexpressing both receptors instead of normal cells with low expression of one or both receptors.

In conclusion, the data of present study evaluated the antitumor efficacy of the bispecific fusion protein Ec-LDP-Hr-AE on esophageal cancers. It displayed extremely potent cytotoxicity to esophageal cancer cells *in vitro*, and caused significant cell cycle arrest and apoptosis. It was also highly effective in inhibiting the growth of KYSE150 xenografts *in vivo*. These results suggested that Ec-LDP-Hr-AE would be a promising candidate for esophageal cancer therapy. Additional studies about the targeting specificity, the biodistribution and metabolism of Ec-LDP-Hr-AE are also required in order to facilitate its clinical application.
